# Qualitative chemical analysis of cinnamon flavored e-cigarette vapor to identify compounds of interest that may affect cardiovascular function

**DOI:** 10.17912/micropub.biology.001605

**Published:** 2025-05-19

**Authors:** Jennifer M. Piechowski, Brian Bagatto

**Affiliations:** 1 Program in Integrated Bioscience, Department of Biology, University of Akron, Akron, Ohio, United States; 2 Department of Biology, Slippery Rock University, Slippery Rock, Pennsylvania, United States

## Abstract

Previous work on the impact of cinnamon flavored electronic cigarette vapor on heart function during early development in zebrafish indicate chemical compounds in the vapor, aside from nicotine, can significantly affect heart function. To determine which compounds were present in the vapor used in our prior study, non-targeted, qualitative gas chromatography/mass spectrometry was performed. Nicotine was found to be present in the nicotine-containing vapor, and it was confirmed to be absent in the nicotine-free vapor. Other chemical compounds that may have affected cardiovascular function in zebrafish were also identified in the analyzed vapors including cinnamaldehyde and eugenol among others.

**Figure 1. Chemical compounds identified in cinnamon flavored, vapor-infused water:**
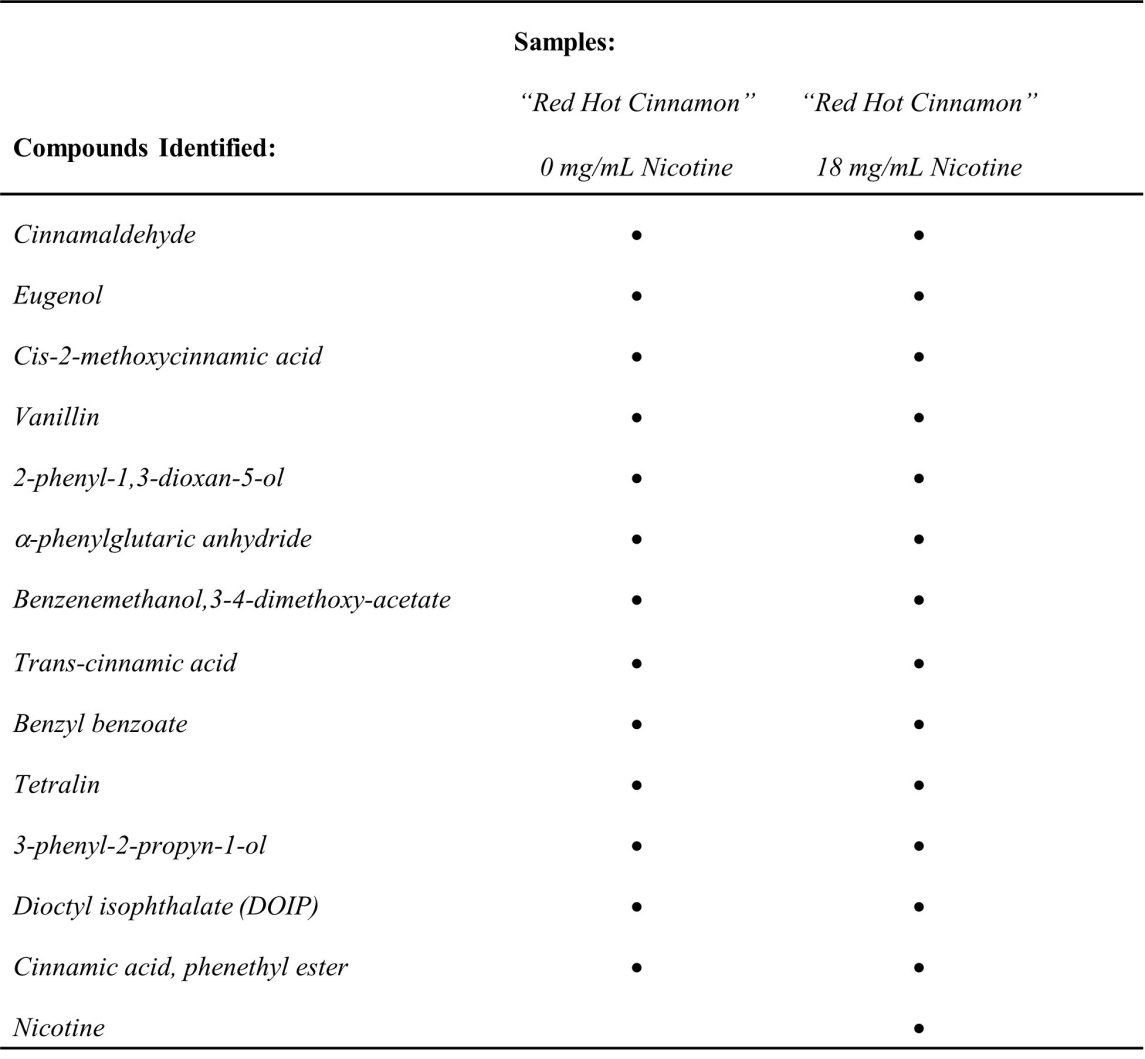
Aggregate, non-targeted, compounds identified in SPE extracted, “Red Hot Cinnamon” flavored, vapor-infused water (with and without nicotine) via qualitative gas chromatography/mass spectrometry analysis.

## Description

It has been previously shown that exposure of zebrafish embryos to nicotine-free cinnamon flavored electronic cigarette (e-cigarette) vapor significantly suppresses cardiovascular function (Piechowski & Bagatto, 2021). Here, qualitative, non-targeted, (Berkelhamer et al., 2019) gas chromatography/mass spectrometry (GC/MS) was performed to confirm the presence of e-cigarette vapor in the zebrafish embryo’s water (thus validating the embryo exposure methods (Piechowski & Bagatto, 2021)) and to identify which chemical compounds were present during the exposures to nicotine-free, cinnamon flavored, e-cigarette vapor.

Compounds identified from the non-targeted, qualitative GC/MS analysis are shown in Figure 1. Through this analysis, it was confirmed that the nicotine-free vaping liquid infused water used previously (Piechowski & Bagatto, 2021) did not contain nicotine when compared to a sample of nicotine containing vaping liquid infused water which was also run as a comparison. Several other compounds of interest including cinnamaldehyde and eugenol were also identified in the vapor-infused water.

The confirmation of the presence of cinnamaldehyde and eugenol may help explain the suppressive cardiovascular effect shown previously with embryo exposures to the nicotine-free, cinnamon flavored vapors (Piechowski & Bagatto, 2021). Eugenol is a primary component of clove oil and is a commonly used fish anesthetic (Grush et al., 2004) that has also been shown to cause suppressive cardiovascular effects in rats (Lahlou et al., 2004), whereas cinnamaldehyde suppresses cardiovascular function by inhibiting voltage gated L-type calcium channels (Alvarez-Collazo et al., 2014). It is vital to recognize that many other compounds in addition to cinnamaldehyde and eugenol were also present in the vapor-infused water and it could be that any, or all, of those compounds may be playing a role in the results shown previously by Piechowski and Bagatto (2021).

More work is needed, however, to examine the effects of nicotine-free, cinnamon flavored vapor with continued zebrafish development as well as further work on exposures to the individual compounds identified here (both individually and in combination) to identify which chemical compounds may affect cardiovascular function throughout development.

## Methods

**Vapor Supplies and Production**

“Red Hot Cinnamon” flavored vaping liquids, labeled as 0 mg/mL and 18 mg/mL nicotine with a 70:30 propylene glycol (PG) to vegetable glycerin (VG) ratio, as well as second-generation all-in-one style vape pens (Joyetech [Shenzhen] Electronics Co., Ltd., Shenzhen, China) were used. E-cigarette vapor was produced from fully-charged, 25 W maximum output second-generation vape pens that were fitted with a cotton wick, 0.6 ohm atomizer head (installed as per manufacturer’s instructions), and filled to the maximum fill line with vaping liquid using methods described previously (Piechowski & Bagatto, 2021). Briefly, vape pens were attached to a 60 mL syringe with fittings, vinyl tubing, and a 3-way valve to allow vapor to be drawn into the syringe before being diverted from the syringe into a 1 L flask filled with 125 mL of dechlorinated water. Flasks were shaken between each puff with each puff lasting 4 seconds (Farsalinos et al., 2013; Gillman et al., 2016; Kennedy et al., 2017; Piechowski & Bagatto, 2021; Spindle et al., 2015). To prevent overheating of the vape pen a 30 second break (Farsalinos et al., 2013; Gillman et al., 2016; Kennedy et al., 2017; Piechowski & Bagatto, 2021) was taken between puffs and tubing was disconnected from the device for 5 minutes after every set of 10 puffs (Piechowski & Bagatto, 2021).

**GC/MS Sample Preparation and Qualitative Analysis**

To provide a concentrated sample of vapor-infused water for compound detection by GC/MS, vapor-infused water samples were prepared at 25 puffs of vapor/125 mL dechlorinated water, where 1 puff = 55 mL vapor (Beauval et al., 2017; Gillman et al., 2016; Kennedy et al., 2017; Mallock et al., 2020; Piechowski & Bagatto, 2021), using the method described above. Samples were analyzed with GC/MS using a non-targeted approach (Berkelhamer et al., 2019) allowing for determination of vapor compounds present in the water. Solid phase extractions (SPE) of the vapor-infused water samples were performed using silica-based SPE cartridges (United Chemical Technologies, LLC) because compounds were being extracted from an aqueous solution. Samples were eluted with methanol (Supleco-Sigma Aldrich, 1998), collected, and delivered to the Mass Spectrometry Lab at The University of Akron for analysis where samples were filtered with a 0.45 μm polytetrafluoroethylene (PTFE) filter prior to being introduced into the GC/MS instrument (Agilent 7820A GC/5975 MSD) via direct, split-less, injection without dilution. The following GC/MS parameters were used: 100 ^o^ C initial temperature; temperature ramped 15 ^o^ C/minute to 150 ^o^ C where samples were held for 2 minutes; temperature ramped 15 ^o^ C/minute to 200 ^o^ C for 2 minute hold; temperature ramped 15 ^o^ C/minute to 250 ^o^ C for 2 minute hold; temperature ramped 15 ^o^ C/minute to 300 ^o^ C with a final 2 minute hold for a total run time of 21.3 minutes per sample. Compounds were identified using the National Institute of Standards and Technology (NIST) Electron-Impact Mass Spectrometry (EI-MS) or Electrospray-Ionization Mass Spectrometry (ESI-MS) database library from 2011 (NIST, 2011a, 2011b).
